# Screening of wild ruminants from the Kaunertal and other alpine regions of Tyrol (Austria) for vector-borne pathogens

**DOI:** 10.1007/s00436-019-06412-9

**Published:** 2019-08-03

**Authors:** Martina Messner, Feodora Natalie Kayikci, Bita Shahi-Barogh, Josef Harl, Christian Messner, Hans-Peter Fuehrer

**Affiliations:** 1 Institute of Parasitology, Department of Pathobiology, University of Veterinary Medicine Vienna, Veterinärplatz 1, 1210 Vienna, Austria; 2 Institute of Pathology and Forensic Veterinary Medicine, University of Veterinary Medicine, Veterinärplatz 1, 1210 Vienna, Austria; 3 Tierarztpraxis Dipl. Tzt. Messner Christian, Alte Landstraße 8a, 6130 Schwaz, Austria

**Keywords:** *Anaplasma phagocytophilum*, Austria, *Babesia capreoli*, Chamois, Ibex, Kaunertal, Tyrol

## Abstract

Knowledge about vector-borne pathogens important for human and veterinary medicine in wild ruminants in Tyrol (Austria) is scarce. Blood samples from Alpine ibex (*Capra ibex*; *n* = 44), Alpine chamois (*Rupicapra rupicapra*; *n =* 21), roe deer (*Capreolus capreolus*; *n =* 18) and red deer (*Cervus elaphus*; *n* = 6) were collected over a period of 4 years (2015–2018) in four regions in North Tyrol, with a primary focus on the Kaunertal. Blood spots on filter paper were tested for the presence of DNA of vector-borne pathogens (*Anaplasmataceae*, Piroplasmida, *Rickettsia* and filarioid helminths). *Anaplasma phagocytophilum* and *Babesia capreoli* were detected in two of 89 (2.3%) blood samples. *Rickettsia* spp., *Theileria* spp. and filarioid helminths were not documented. One Alpine chamois was positive for *A. phagocytophilum* and *B. capreoli*. Moreover, an ibex from the Kaunertal region was positive for *A. phagocytophilum*. While the ibex was a kid less than 1 year old, the chamois was an adult individual. Further research is recommended to evaluate effects of climate change on infection rates of North Tyrolean wild ruminants by these pathogens and the distribution of their vectors.

## Introduction

Wild ruminants such as red deer (*Cervus elaphus*), roe deer (*Capreolus capreolus*) and chamois (*Rupicapra rupicapra*) inhabit major parts of the Tyrolean Alps. While in the past, these naturally elusive animals had scarcely any points of contact with humankind, the opening of mountain areas and the increased use of alpine regions by humans has blurred the boundaries between civilisation and wildlife. This development allows transmission of pathogens between wild ruminants and grazing livestock, often from domesticated to wild animals (Giacometti et al. 2002; Rossi et al. 2007), and vice-versa (Bischofberger and Nabholz 1964; Chintoan-Uta et al. 2014). Apart from the role of wild ungulates as a direct source of infection, they have been shown to act as wildlife reservoirs for several causative agents of disease relevant for human and veterinary medicine (Hoby et al. 2009; Gelormini et al. 2016; Michel et al. 2014). Some of the most significant medical conditions that affect wild ruminants are vector-borne diseases, which include protozoa such as *Babesia* spp. and bacteria such as *Anaplasma* spp. These pathogens are transmitted by ticks, which are ubiquitous in lowland regions of Europe, while their prevalence decreases at higher elevations (Boehnke et al. 2015). Of all the indigenous ruminants, the one inhabiting the highest altitudes and therefore probably the least likely to be infected by tick-borne diseases is the Alpine ibex (*Capra ibex*). This caprine, which almost became extinct in the nineteenth century, was reintroduced successfully to the Alpine landscape and is now indigenous to many European countries, with Switzerland, Italy and France harbouring the vast majority of ibex populations. In Tyrol, Austria, the ibex population was reduced to zero in the second half of the seventeenth century and reintroduced in the twentieth century, with the valleys Pitztal and Kaunertal playing a vital role (Bauer 2016). In 2015, a total number of 4992 ibex were recorded in Tyrol (Rudigier 2015), with rising tendency. The ibex occupies the area between the tree line and the snow line in summer and descends to lower territories in winter. Though its natural habitat makes it the least likely to come into contact with livestock or even vectors such as ticks, ibex have not only been shown to be susceptible for infections with for instance *Anaplasma phagocytophilum* or *Babesia capreoli*, incidences of infections with these pathogens have also been previously documented in Tyrol (Silaghi et al. 2011a). In this study, we aimed to assess the presence of these vector-borne pathogens in a larger number of Alpine ibex and other wild ruminants from this region.

## Materials and methods

From November 2015 to February 2018, a total of 89 blood samples were collected from different species of wild ungulates, namely Alpine ibex (*Capra ibex*, *n* = 44), Alpine chamois (*Rupicapra rupicapra*, *n* = 21), roe deer (*Capreolus capreolus*, *n* = 18) and red deer (*Cervus elaphus*, *n* = 6). A majority of the samples were taken from game during the hunting season (August to December), others from perished animals. The tested animals originated from four regions in the North Tyrolean Alps: Kaunertal, Achental, Stanzertal and Paznauntal.

This study mainly focuses on the Kaunertal, a region in the southwest of North Tyrol, belonging to the Ötztaler Alps. Its highest peak, the Weißseespitze (3501 m), is part of Austria’s second largest glacier, Gepatschferner, which borders Italy. All of the hunting grounds lie above 1300 m of altitude. The Stanzertal and the Paznauntal, a side valley of the Stanzertal, are located northwest from the Kaunertal, close to Vorarlberg and Switzerland. The samples taken in these regions stem from the Verwall Group which reaches altitudes similar to the mountains of the Kaunertal, ranging from 1300 to 3167 m. The Achental lies in the north of North Tyrol near the Bavarian border and belongs to the Karwendel Mountains. The animals tested from this area populated heights between 900 and 2100 m above sea level, thereby reaching lower altitudes than the animals from other regions.

Filter paper (Whatman® FTA® Elute Cards) was used for the collection of the blood samples which were stored at room temperature until further processing. DNA extraction from the blood spots was performed as reported previously (Fuehrer et al. 2010). After soaking the blood spots in 100 ml of phosphate-puffered saline at 4 °C overnight, the DNA was isolated and purified using InstaGene™ Matrix (Bio-Rad, Austria). Afterwards, it was amplified by polymerase chain reaction (PCR) to detect infections with piroplasms, *Anaplasmataceae*, *Rickettsia* spp., and filarioid nematodes using established protocols (Table 1). PCR products were analysed by electrophoresis in 2% agarose gels stained with Midori Green Advance DNA stain (Nippon Genetics Europe, Germany). Positive samples were sent to a commercial company (LGC Genomics GmbH, Germany) for sequencing.

**Table 1:** Primer sequences, targeted genes and protocol references used in this study (modified table after Hodžić et al. (2015))

Target organism (genetic marker)	Primer sequences (5′ → 3′)	Product size (bp)	Reference
Anaplasmataceae (16S rRNA)	EHR16SD: GGTACCYACAGAAGAAGTCC EHR16SR: TAGCACTCATCGTTTACAGC	345	Brown et al. (2001)
*A. phagocytophilum* (groEL)	Apgroe02for: CGAAAGCTGCTGGATCTGA Aprgroe02rev: TCCTTGAAGCCTTTGCTTTC	235	Hodžić et al. (2018)
*Babesia* spp. (18S rRNA)	BTH-1F: CCTGAGAAACGGCTACCACATCT BTH-1R: TTGCGACCATACTCCCCCCA	700	Zintl et al. (2011)
	Nested PCR GF2: GTCTTGTAATTGGAATGATGG GR2: CCAAAGACTTTGATTTCTCTC	561	
Filarioid nematodes (CO1)	H14FilaCO1Fw: GCCTATTTTGATTGGTGGTTTTGG H14FilaCO1Rv: AGCAATAATCATAGTAGCAGCACTAA	724	Hodžić et al. (2015)
*Rickettsia* spp. (23S/5S rRNA)	ITS-F: GATAGGTCGGGTGTGGAAG ITS-R: TCGGGATGGGATCGTGTG	350–550	Vitorino et al. (2003)

In order to visualize the geographic and host distribution of the *Babesia* lineage isolated from *R. rupicapra* (sample GF12), we calculated a median-joining haplotype network including sequences published on GenBank. We performed a BLAST search against NCBI GenBank with the *18S* sequence of “sample ID” and downloaded all sequences showing a minimum of 97% similarity. We retrieved a total 157 sequences of which 37 were shorter than the query sequence and ten sequences were excluded because they contained ambiguities or obvious sequencing errors. Information on host species and country/region were either extracted from the GenBank files or the respective publications. A median joining network was calculated in Network v.5.0.1.0 (Fluxus Technology, Suffolk, England) with the 469 bp alignment containing the remaining 109 sequences plus that of sample GF12. Information on the host species and the geographic region was added in Network Publisher v.2.1.2.3 (Fluxus Technology), and the network was graphically prepared in Adobe Illustrator CC v.19.0.0 (Adobe Inc., San José, California, USA).

## Results and discussion

The animals were divided into age groups of kids/fawns (<1 year), young (1–4 years), middle (5–9 years) and old aged (>9 years) animals (information was provided by the hunters). Four animals were assigned to the first, 14 to the second, 17 to the third and 43 to the fourth category. In 11 cases no age statement was provided. Forty-seven of the animals sampled were male, 41 female, and in the case of one kid, the sex was not determined. Table 2 shows a detailed overview of the numbers of samples taken from each species associated with the corresponding location.

**Table 2:** Number and origin of blood samples taken from wild ungulates in the North Tyrolean Alps from 2015 to 2018

	Chamois	Ibex	Red deer	Roe deer	Total
Total no. of tested animals (no. of positive animals)					
Achental	7 (1)	7 (0)	0 (0)	0 (0)	14 (1)
Kaunertal	13 (0)	35 (1)	6 (0)	18 (0)	72 (1)
Patznauntal	0 (0)	1 (0)	0 (0)	0 (0)	1 (0)
Stanzertal	1 (0)	1 (0)	0 (0)	0 (0)	2 (0)

From a total of 89 blood samples, two (2.3%) tested positive for pathogen DNA. One was in a chamois, the other from an ibex. The chamois originated from the Karwendel region and was infected with *A. phagocytophilum* and *B. capreoli* (isolate: GF12; GenBank, MK982248). This particular animal was found in an immobilized and moribund state and had to be shot by a huntsman. A subsequent dissection revealed it to suffer from severe anaemia, haemorrhagic ascites as well as infections with *Haemonchus contortus* and *Fasciola hepatica*. Fatal cases of *B. capreoli* infections in chamois have been reported previously (Hoby et al. 2007). The *16S* sequences of isolates of *A. phagocytophilum* were identical with sequences obtained from cattle (e.g. KJ832476) and roe deer (e.g. KJ832453) from France, a chamois from Slovenia (KM215265), an elk from Sweden (KC800986) and a roe deer from Austria (e.g. AY220467) among others.

**Fig. 1:**
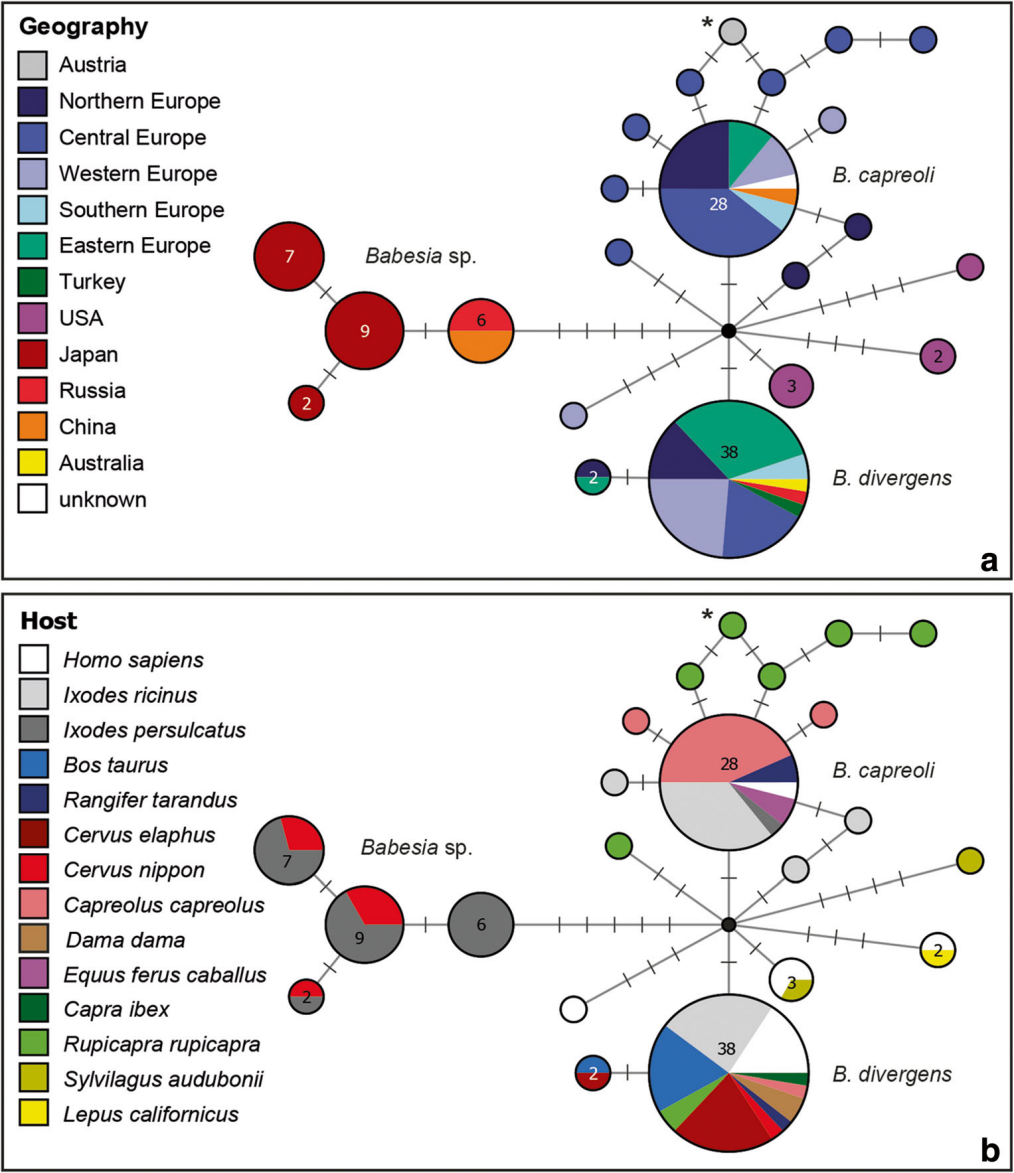
Median-joining haplotype network of *Babesia divergens* and related *18S* lineages: **a** Geographic distribution and **b** host distribution of genotypes of *Babesia capreoli* and related lineages. The number of samples featuring the same genotype is indicated for every haplotype containing more than one sequence. The bars on the lines indicate the number of substitutions between two haplotypes. The black node represents an intermediate, unobserved node. The asterisk indicates the genotype GF12 from the present study

The *B. capreoli* sample GF12 featured a new *18S* genotype differing in one nucleotide position from two genotypes isolated from *R. rupicapra* in Tyrol, Austria (Fig. 1; JN543172 and JN543177; Silaghi et al. 2011a). Two other genotypes, differing in two and three nucleotides from sample GF12, were isolated from *R. rupicapra* in Tyrol (JN543181; Silaghi et al. 2011a) and Switzerland (EU182596; Schmid et al. 2008), respectively. Sample GF12 also differs in two nucleotide positions from the main genotype attributed to *B. capreoli*, which was isolated from *C. capreolus* (12 individuals), *Ixodes ricinus* (10), *I. persulcatus* (1), *Rangifer tarandus* (2), *Equus ferus caballus* (2) in Europe, and a human in China (MK256977; published on GenBank only). Five other genotypes differing in one or two nucleotide positions from the main *B. capreoli* genotype were isolated from *C. capreolus* (AY572456, GQ304526) and *I. ricinus* (KF447531, MH351710, MH351711) in Europe. The most frequent *B. capreoli* genotype differs only in two nucleotide positions from the most frequent *B. divergens* genotype. The network also features a cluster with four similar genotypes isolated from *C. nippon* in Japan and *I. persulcatus* in Japan, China and Russia. These genotypes were classified as *B. divergens*, but differ in six or more nucleotide positions from the latter. Three other genotypes were isolated from rabbits and humans in the USA and probably also belong to different *Babesia* species.

The infected ibex was from the Kaunertal and was found to be positive for *A. phagocytophilum*. It was less than a year old and thus the youngest ibex examined in this study. None of the older ibexes was tested positive. In a previous study conducted in Tyrol in 2011, *A. phagocytophilum* was detected in all species tested (Silaghi et al. 2011a). In the latter study, a total of 53 animals were tested, including 35 from the Karwendel and 12 from the Kaunertal. Two out of seven studied ibex and four out of 10 chamois were positive. While there were positive results for members of all species from the Karwendel, none of the animals from the Kaunertal carried pathogen DNA (Silaghi et al. 2011a). Our results show that at least one ibex from the Kaunertal region was infected with *A. phagocytophilum*. Overall, the prevalence of the studied pathogens still appears low in this area. The ibexes tested from the Karwendel, on the contrary, were all found to be free of pathogen infection.

A high prevalence of pathogen DNA in roe deer has been reported in Austria and Tyrol’s neighbouring countries, namely 18.4% (Switzerland; Liz et al. 2002), 23.9% (Switzerland; Michel et al. 2014), 31% (Italy; Tampieri et al. 2008) and 85.9% (Germany; Overzier et al. 2013) for *Babesia* spp*.* and 47.1% (Austria; Silaghi et al. 2011a), 54.2% (Italy; Di Domenico et al. 2016) and 83.2% (Germany; Kauffmann et al. 2017) for *A. phagocytophilum* respectively. Prevalences in red deer were 5.1% (Austria; Cézanne et al. 2017), 17.3% (Switzerland; Michel et al. 2014) and 26.7% (Italy; Ebani et al. 2016) for *Babesia* spp. and 1.5% (Austria; Cézanne et al. 2017), 40% (Italy; Ebani et al. 2016), 70% (Austria; Silaghi et al. 2011a) and 75% (Italy; Di Domenico et al. 2016) for *A. phagocytophilum*. In the present study, no roe deer or red deer tested positive for pathogen DNA.

Although there are studies which suggest otherwise (Liz et al. 2002; Ebani et al. 2016), age seems to act as a significant factor for infection with *A. phagocytophilum*, with animals younger than 3 years being more susceptible to contagion (Silaghi et al. 2011a, b). This is in accordance with the fact that the positive ibex in this study was less than 1 year old. *B. capreoli* infections have also shown a positive correlation with young age (Michel et al. 2014). For the infected chamois in the present study, no age class was specified, but it was at least 3 years of age or older. In previous studies, no correlation between the host’s sex and infection with both *B. capreoli* and *A. phagocytophilum* was found (Liz et al. 2002; Silaghi et al. 2011a; Michel et al. 2014; Ebani et al. 2016; Cézanne et al. 2017). Both animals positive for pathogen DNA in this study were female.

The occurrence of ticks in North Tyrol (*Ixodes ricinus* being the main representative in Central Europe) has been previously documented at elevations up to more than 1500 m above sea level (Sonnleitner et al. 2015), but benefitting from environmental changes, the distribution of the ticks now reaches a higher elevation. In the Kaunertal’s neighbouring valley Ötztal, *I. ricinus* has been located at altitudes of 2000 m, and altitudes of 1700 m are evidenced in the entire state (personal communication, Gernot Walder). During the warm months, when ticks are most active, the ibex tend to stay at higher elevations, whereas they descend in winter when the tick’s activity and thus the threat of infection decrease significantly. While it is possible that the kid contracted *A. phagocytophilum* during its migration to lower altitudes, an alternative cause could be that the ticks reach much higher altitudes than presumed. It might, therefore, be interesting to evaluate the height above sea level that ticks carrying *A. phagocytophilum* reach. *I. ricinus*, populating considerably lower elevations, have previously been reported to show prevalences of *A. phagocytophilum* of up to 8.9% in Bavaria (Overzier et al. 2013) and 1.8% in South Tyrol (Baráková et al. 2014). Since wild ungulates populating the Achental reach lower altitudes than those from the Kaunertal, the animals from the Karwendel would be assumed to have higher infection rates of *A. phagocytophilum*, as reported by Silaghi et al. 2011a, b. *I. ricinus* might be favoured due to lower elevation of the Karwendel mountains (Silaghi et al. 2011b). The widely differing numbers of samples taken from both regions as well as the small sample size from the Achental does not allow for direct comparison of this study’s findings in this context. Likewise, the number of samples from the Stanzertal and the Paznauntal were far too small to draw conclusions from the corresponding results and leave room for further investigation. Increased occurrence of *B. capreoli* is linked to low altitudes as well, as shown by Michel et al. 2014. Interestingly, *B. capreoli* was not detected in roe deer but chamois, although roe deer show a higher incidence for *B. capreoli* infections (Hoby et al. 2009; Michel et al. 2014). Coinfections of *B. capreoli* and *A. phagocytophilum*, such as in the present case, are common in wild ungulates in Austria (Silaghi et al. 2011b).

Despite the low incidence of infected animals found in this study, the advancing distribution of vectors into higher Alpine regions suggests that continuous monitoring of these pathogens in wild ruminants would be desirable.
